# Overcoming Vaccine Inequities and Research Gaps in Africa: Challenges and Opportunities Identified During the COVID-19 Pandemic

**DOI:** 10.1093/cid/ciaf055

**Published:** 2025-07-22

**Authors:** Cara Lynn Kim, Thaint Thaint Thwe, Ligia M Cruz Espinoza, Jonathan D Sugimoto, Mosoka P Fallah, Hyon Jin Jeon, Raphaël Rakotozandrindrainy, Ellis Owusu-Dabo, Abdramane Soura Bassiahi, Octavie Lunguya, Ilesh V Jani, Florian Marks, Birkneh Tilahun Tadesse

**Affiliations:** International Vaccine Institute, Seoul, Republic of Korea; International Vaccine Institute, Seoul, Republic of Korea; International Vaccine Institute, Seoul, Republic of Korea; International Vaccine Institute, Seoul, Republic of Korea; Department of Epidemiology, University of Washington, Seattle, Washington, USA; Research and Innovation, Africa Centers for Disease Control, Addis Ababa, Ethiopia; International Vaccine Institute, Seoul, Republic of Korea; Cambridge Institute of Therapeutic Immunology and Infectious Disease, University of Cambridge School of Clinical Medicine, Cambridge Biomedical Campus, Cambridge, United Kingdom; Madagascar Institute for Vaccine Research, University of Antananarivo, Antananarivo, Madagascar; Madagascar Institute for Vaccine Research, University of Antananarivo, Antananarivo, Madagascar; School of Public Health, Kwame Nkrumah University of Science and Technology, Kumasi, Ghana; Institut Supérieur des Sciences de la Population, Université Ouaga II, Ouagadougou, Burkina Faso; Department of Microbiology, Institut National de Recherche Biomédicale, Kinshasa, Democratic Republic of the Congo; Department of Medical Biology, Microbiology Service, University Teaching Hospital of Kinshasa, University of Kinshasa, Kinshasa, Democratic Republic of the Congo; Instituto Nacional de Saúde, Maputo, Mozambique; International Vaccine Institute, Seoul, Republic of Korea; Cambridge Institute of Therapeutic Immunology and Infectious Disease, University of Cambridge School of Clinical Medicine, Cambridge Biomedical Campus, Cambridge, United Kingdom; Madagascar Institute for Vaccine Research, University of Antananarivo, Antananarivo, Madagascar; Heidelberg Institute of Global Health, University of Heidelberg, Heidelberg, Germany; The Hong Kong Jockey Club Global Health Institute, Hong Kong Special Administrative Region, China; International Vaccine Institute, Seoul, Republic of Korea; Heidelberg Institute of Global Health, University of Heidelberg, Heidelberg, Germany; Department of Global Public Health, Karolinska Institutet, Stockholm, Sweden

**Keywords:** vaccine inequity, COVID-19 pandemic, Africa, pandemic response, vaccine research and development

## Abstract

Before the onset of the coronavirus disease 2019 (COVID-19) pandemic, the African region already faced a substantial communicable disease burden and a rising prevalence of noncommunicable and chronic conditions. The COVID-19 pandemic has underscored critical vulnerabilities within the health systems in African countries and highlighted the urgent need for self-sufficiency to increase resilience in vaccine manufacturing and clinical trial capacity. In response, substantial efforts are underway to develop vaccine and pharmaceutical manufacturing capabilities across the continent. Ongoing initiatives supported by large donor organizations are aimed at initiating much-needed progress toward greater self-sufficiency in vaccine production in Africa, as coordinated by the Africa Centers for Disease Control. Continued investment, regulatory harmonization, and strengthened international and regional partnerships are essential for Africa to develop sustainable vaccine manufacturing.

The global effects of the unprecedented coronavirus disease 2019 (COVID-19) pandemic have substantially varied across countries and regions. Although Africa appeared to be less severely affected by the pandemic than many high-income regions in terms of disease burden, it faced unique challenges arising from its less-developed public health infrastructure and high endemic disease burden [1, 2].

Services, such as immunization, family planning, antenatal care, and management of prevalent communicable diseases, were adversely affected. According to UNICEF and World Health Organization (WHO) estimates, approximately 7.7 million children in Africa did not receive the first doses of routine immunizations for diphtheria-tetanus-pertussis, polio, and measles in 2020, in addition to several million who did not receive other routine immunizations and follow-up doses. Although immunization programs resumed, and increased coverage was reported in 2022, coverage rates still remain below 2019 pre-pandemic levels [3, 4]. The Global Fund to Fight AIDS, Tuberculosis, and Malaria has reported that 2020 was the first year since its initiation in which key progress indicators declined [5]. Furthermore, the exacerbation of these prevalent communicable diseases in sub-Saharan Africa markedly exceeded the effects of COVID-19 itself, as measured by mortality and disability-adjusted life-years [6]. Similarly, the detection and treatment of several neglected tropical diseases, such as dengue, Lassa fever, leprosy, and leishmaniasis, were also hampered during the pandemic [7–9].

With regard to COVID-19 burden, the number of cases and observed mortality rates in Africa were lower than those in other regions globally. However, several studies have suggested substantial underreporting and inaccuracy in recorded COVID-19 cases and deaths within sub-Saharan Africa. The WHO African region reported 7.1 million cumulative cases by the end of 2021 (Figure 1), whereas a modeling study estimated that 505.6 million people had been infected by that time [11]. Seroprevalence studies in 2021 have also indicated that a much higher proportion of the African population had been exposed to severe acute respiratory syndrome coronavirus 2 (SARS-CoV-2) compared to previous surveillance data, with an estimated overall seroprevalence of 65.1% [12]. Research on mortality rates has indicated that only 35.3% of the estimated 0.5 million deaths were reported as COVID-19–related deaths [11]. Another large-scale study on excess mortality in 74 countries has estimated an excess mortality rate of 101.6 per 100 000 in sub-Saharan Africa, which is comparable to the 125.8 per 100 000 excess mortality rate estimated for high-income countries [13].

**Figure 1.**
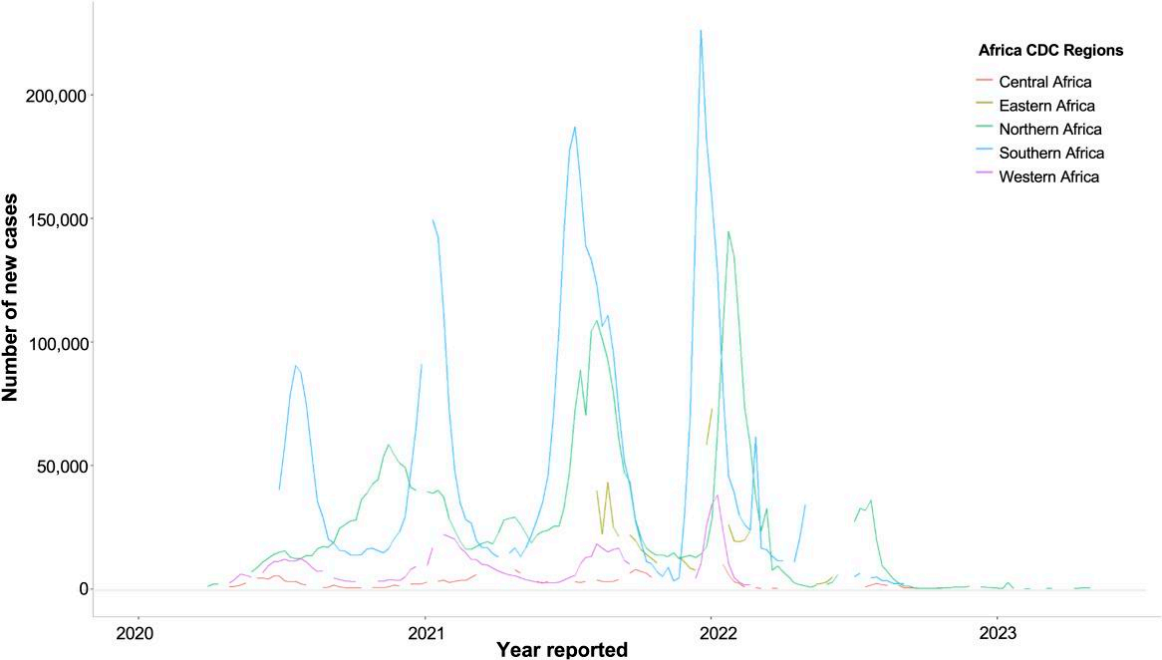
Number of new COVID-19 cases per African CDC region. Figure created using data from reference 10. Abbreviations: Africa CDC, Africa Centers for Disease Control and Prevention; COVID-19, coronavirus disease 2019.

In many African countries, reduced testing and reporting capacity might have contributed to regional disparities in the number of daily new COVID-19 cases across Africa (Figure 1) [14, 15]. Southern and Northern Africa experienced high daily case numbers throughout the pandemic, while Central Africa and Western Africa reported lower case numbers; meanwhile, Eastern Africa had substantial missing data. These limitations, coupled with disruptions in healthcare systems—including Africa's importation of medicines, diagnostic equipment, and vaccines from overseas during the pandemic—highlight the continent's vulnerabilities and dependency on global markets [7, 16–18].

Nevertheless, Africa made substantial advancements in surveillance and public health infrastructure during the pandemic, benefiting from the collaboration among African Union members and the Africa Centers for Disease Control (Africa CDC) established in response to previous Ebola outbreaks [19]. The lessons learned from those previous outbreaks proved invaluable during the COVID-19 pandemic, in enhancing the detection, testing, and control capacities of many countries on the continent. The coordination and collaboration fostered by these epidemics and the COVID-19 pandemic have enabled Africa to optimize its available resources and have demonstrated its strength in translational research and vaccine development. This article is aimed at emphasizing the importance of accelerating translational research and development in African countries to improve health security on the continent, particularly in light of the disparities revealed by the COVID-19 pandemic, and to discuss current efforts toward achieving this goal.

## COVID-19 RESEARCH AND CLINICAL TRIALS IN AFRICA

Beyond the underreporting of COVID-19 disease burden and vulnerabilities within healthcare systems in Africa, the pandemic increased awareness of global inequalities in research and vaccine development. Even before the COVID-19 pandemic, only 2% of clinical trials worldwide were conducted in Africa [20]. Despite being a home to more than 1 billion people, and bearing more than 20% of the world's overall disease burden, including a substantial proportion of global malaria cases, Africa has been disadvantaged by limited clinical research data, thus restricting the applicability of findings to the region's specific health needs [21, 22].

In contrast to many high-income countries, there was a lack of real-world data reporting from most African countries assessing the effects of the pandemic between 2020 and 2023. Many published data have relied on modeling studies and estimation. Clinical trials, such as “Expanding Access and Delivery of COVID-19 Vaccines in Africa (ECOVA): Effectiveness Evaluation of the Sinopharm Vaccine in the Dondo District in Mozambique,” arising from collaborations between African research institutions and the International Vaccine Institute (IVI), represent crucial efforts in addressing gaps in real-world data on vaccine effectiveness, including the studies presented in this supplement in page S37 and S47 [23–25]. By August 2020, among more than 36 000 COVID-19–related publications, only 3% were authored by Africans, predominantly from South Africa, Egypt, and Nigeria [26]. By 2021, only 1% of COVID-19–related research in Africa focused on therapeutics and vaccines, despite a global surge in vaccine research [27, 28]. Clinical research, including vaccine trials, has been concentrated in several countries, such as Kenya, Ghana, Egypt, and South Africa, thereby limiting the applicability of results across Africa, a region characterized by geographic heterogeneity [29–31]. This discrepancy is likely to be associated with pre-existing disparities in research capacity and infrastructure associated with economic inequalities. For instance, 54% of COVID-19 vaccine trials were conducted in South Africa, which has often been the only African country to participate in large multinational trials [29, 30]. This geographic imbalance is reflected in the gross domestic expenditure on research and development, often expressed as a percentage of the Gross Domestic Product and used as a proxy for investment in research and development: South Africa leads, at 0.82%, yet, this percentage is significantly lower than those in Asia and Europe, which each invest approximately 30%. The average across sub-Saharan Africa is only 0.4%, thus, highlighting the stark differences in research investment across the continent [32, 33].

Investment in COVID-19 research and clinical trials in Africa has also been sparser than that in the rest of the world. In July 2020, Africa received only 3% (US$22 million) of global COVID-19 research funding, and 4.5% of projects (84 of 1858) were based in African countries, with funding primarily from European and US institutions. Despite an increase in global investment during 2022, Africa's share rose to only 4%, with US$267 million and 786 of 17 955 research projects [34]. Nonetheless, many trial sites in Africa are part of multisite or multinational trials and receive funding from outside the continent [29]. By October 2021, Africa accounted for 4% of all COVID-19–related clinical studies listed on the International Clinical Trial Registry Platform, as well as 7% of COVID-19 vaccine trials by May 2022 [29, 35]. The Pan African Clinical Registry Platform (https://pactr.samrc.ac.za/), established in 2007, lists a number of COVID-19 vaccine clinical trials, but many have not yet started enrollment, lack ethical approval, or have not been updated since their approval, thereby highlighting ongoing challenges and heterogeneities in the clinical trial landscape across the continent.

Conducting clinical trials in low- and middle-income countries (LMICs) poses major challenges, including limited financial and human resources, ethical and regulatory constraints, and inadequate research infrastructure [36]. Nevertheless, LMICs, including those in Africa, bear substantial disease burdens that are inadequately studied in high-income countries.

## PERSISTING INEQUALITIES: VACCINES AND VACCINATION

**Figure 2.**
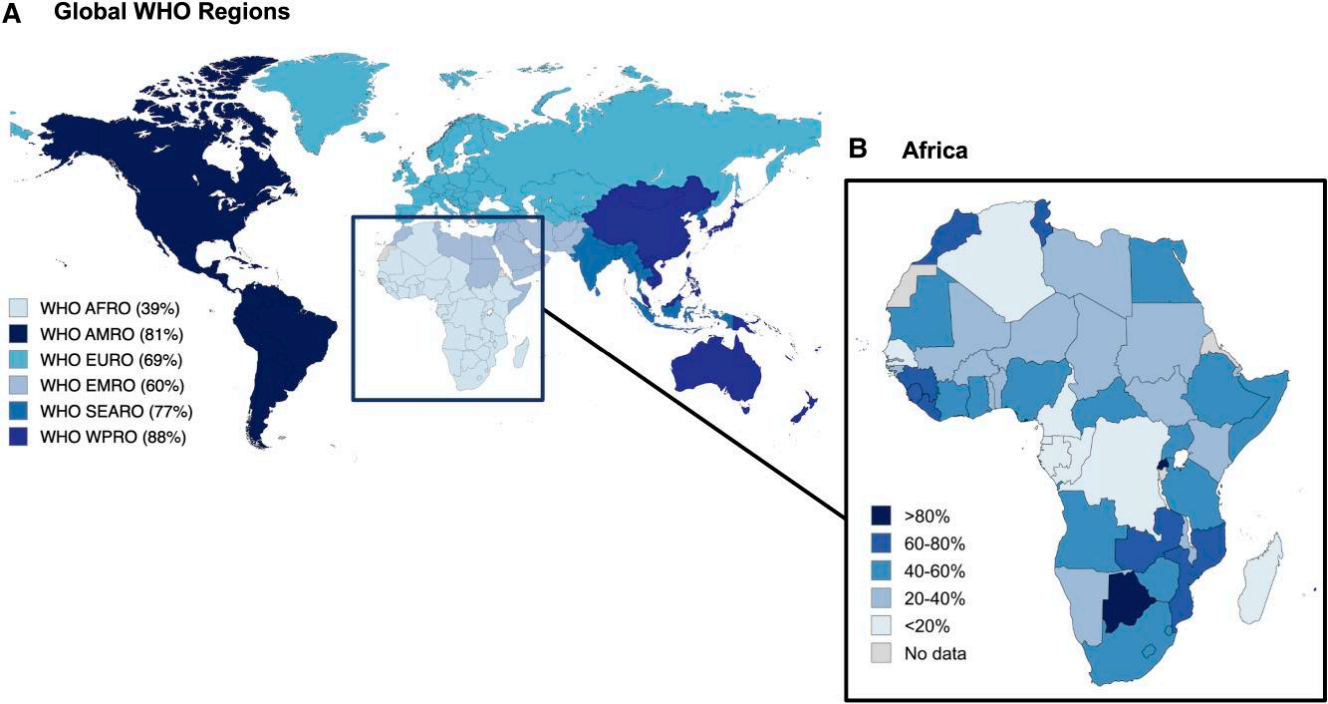
*A* and *B*, Percentage of total population vaccinated with at least one dose of a COVID-19 vaccine by December 2023. Figure created using data from reference 39. Abbreviations: AFRO, African Region; AMRO, Region of the Americas; COVID-19, coronavirus disease 2019; EMRO, Eastern Mediterranean Region; EURO, European Region; SEARO, South-East Asian Region; WHO, World Health Organization; WPRO, Western Pacific Region.

**Figure 3.**
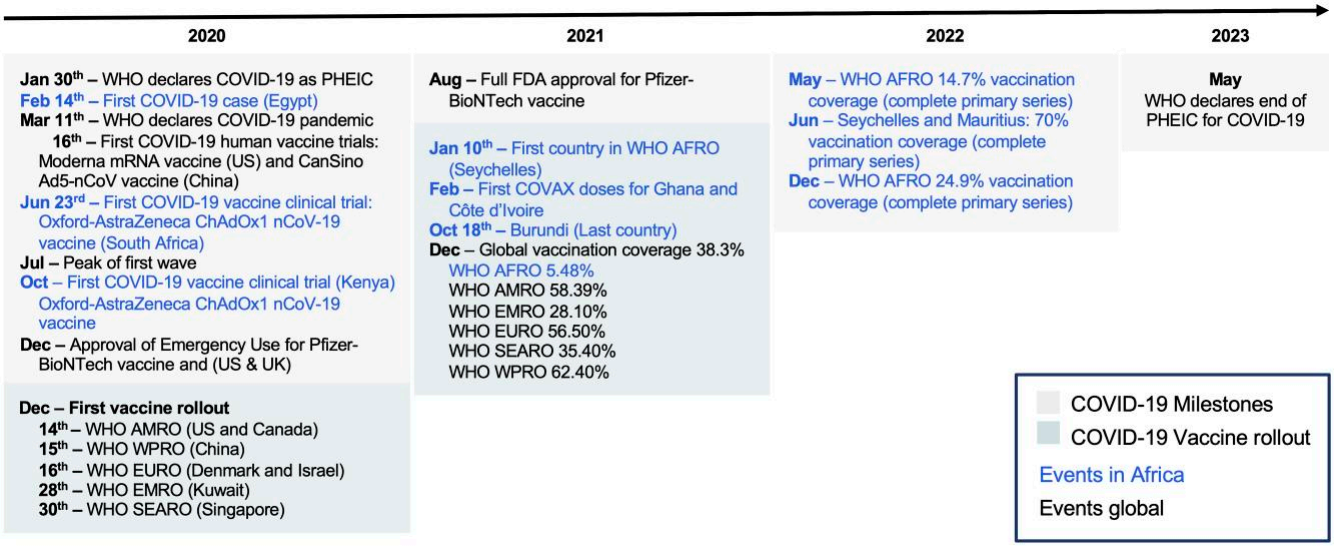
Timeline of COVID-19 events. Figure was created using data from references 10, 43–49. Abbreviations: AFRO, African Region; AMRO, Region of the Americas; COVAX, COVID-19 Vaccines Global Access; COVID-19, coronavirus disease 2019; EMRO, Eastern Mediterranean Region; EUA, Emergency Use Authorization; EURO, European Region; FDA, Food and Drug Administration; PHEIC, Public Health Emergency of International Concern; SEARO, South-East Asia Region; WHO, World Health Organization; WPRO, Western Pacific Region.

The COVID-19 pandemic led to an unprecedented surge in global collaboration, which accelerated vaccine development and distribution [37]. However, on the African continent, the COVID-19 vaccine rollout highlighted substantial inequalities. According to the WHO's global vaccination coverage data, 67% of the total population had received a complete primary vaccination series, and 32% had received a booster dose, by December 2023 (Figure 2) [38]. In contrast, the WHO African Region reported much lower rates, with 39% of the population having received at least one dose, 33% having completed the primary series, and only 6% having received a booster [40]. These data highlight that the African region has the lowest vaccination coverage worldwide; the second-lowest rates were recorded in the WHO Eastern Mediterranean Region, with 60%, 52%, and 19% for coverage for at least one dose, complete primary series, and booster dose, respectively [41]. Meanwhile, the first-dose coverage rate exceeded 80% in the WHO Region of the Americas and Western Pacific Region, and was approximately 77% in the WHO Southeast Asia Region [42]. The data further indicated a delay in vaccine rollout: African nations were among the last to start vaccination campaigns (Figure 3). Vaccination campaigns began in most parts of the world in December 2020, whereas the first national vaccination campaigns on the African continent were initiated in January 2021, in the Seychelles, and many African countries started their campaigns in the following months [50, 51]. In February 2021, the first COVID-19 Vaccines Global Access (COVAX) vaccine doses were delivered to Ghana and Côte d’Ivoire, in the largest immunization drive to date, thus, demonstrating global efforts to increase COVID-19 vaccine accessibility [52, 53]. By December 2024, more than 60% of vaccines in Africa, approximately 713 million doses, had been delivered through COVAX, thereby showcasing the importance of this scheme for Africa's vaccination drive [54].

Importantly, discrepancies exist between the data from the WHO and the Africa CDC: the latter publishes more up-to-date figures, and the WHO African region does not include countries such as Egypt, Morocco, Somalia, Sudan, Tunisia, and Djibouti. Nevertheless, both sources highlight additional discrepancies within the continent: only three countries—Botswana, Rwanda, and the Seychelles—achieved first-dose coverage above 80%, whereas several countries remained below 20%, including Algeria, Cameroon, the Democratic Republic of the Congo, and Senegal. Data for booster vaccination are limited, and only 35 African member states offered booster doses [55].

Limited vaccine supply remained a major obstacle to vaccination, particularly in the first year of the pandemic. By November 2021, the four major pharmaceutical companies in Europe and North America (Moderna, AstraZeneca, Pfizer/BioNTech, and Johnson & Johnson) had not been able to allocate more than 25% of their vaccine supply to COVAX, as originally committed [56]. Despite the increasing number of vaccine doses shipped to Africa, with 449 million doses delivered in 2022 (a 40% increase over the previous year), persistent challenges in vaccine deployment have continued to hinder effective vaccination. In 2021, 53% of delivered doses were administered; in 2022, this percentage increased to 70% of received doses, representing approximately 540 million administered doses [57, 58]. A lack of resources, limited infrastructure for storage and distribution, and vaccine hesitancy have been identified as factors reducing vaccine uptake [58, 59]. More than 21 million expired doses, or 3.0% of doses received in the African Region, were reported by December 2022; Madagascar, Algeria, and the Democratic Republic of the Congo reported that more than 20% of their vaccine doses had expired [60]. These data reveal a heterogeneous vaccine rollout and substantial disparities in vaccine distribution, availability, and readiness in Africa as compared with other continents, as well as across African nations. This underscores the urgent need for increased investment in vaccine manufacturing, delivery, distribution, and administration to achieve sustainable vaccination progress.

Although the current landscape of clinical research in Africa is modest, efforts made during the COVID-19 pandemic could accelerate future research activities on the continent. Building research capacity in collaboration with African governments has occurred at multiple occasions during the pandemic, thereby advancing the local development of diagnostics and therapeutic agents specifically suited to its population's needs, and paving the way to sustainable improvements in digital infrastructure [61]. Africa's potential as a future hotspot for clinical research has been attributed to its large and highly genetically diverse population that experiences high rates of both communicable and noncommunicable diseases [62]. Similar to how research conducted during the Ebola outbreak helped build local infrastructure and capacities, COVID-19 research in Africa could provide a foundation for vaccine manufacturing—for example, through improved clinical trial infrastructure, locally operated laboratory facilities, and strengthened collaborations [63, 64].

## ACCELERATING TRANSLATIONAL RESEARCH AND DEVELOPMENT IN AFRICA

The COVID-19 pandemic exposed unmet needs, gaps, and challenges in health systems worldwide. The pandemic also increased global awareness of the critical needs for effective infectious disease management, resilient healthcare systems, sustainable health solutions, and international scientific collaborations. In Africa, this renewed focus underscores the needs for investing not only in research and development for self-reliance but also in strengthening health institutions, developing early warning systems, and establishing centralized governance structures. Such investments are considered essential for ensuring both the health security and economic stability of the continent [19].

The expansion of vaccine and pharmaceutical manufacturing presents major opportunities for the African continent, and both the WHO and its Member States have begun leveraging Africa's potential to enhance local manufacturing capabilities. In 2021, only 1% of COVID-19 vaccines were manufactured locally in Africa, and the continent was heavily reliant on imports, primarily from India. At that time, approximately 10 vaccine manufacturers were established or planned across Africa, in countries including South Africa, Ethiopia, Egypt, Tunisia, Nigeria, Ghana, and Senegal. However, geographic disparities remain, particularly in Central Africa, where vaccination rates are among the lowest in Africa [39, 65]. To address these challenges and improve self-sufficiency in the region, the Africa CDC has set a goal of producing 60% of Africa's required vaccines locally by 2040 [66, 67]. Achieving this goal will require sustained investments and global collaborations.

In May 2024, BioNTech and The Coalition for Epidemic Preparedness Innovations (CEPI) announced an expansion of their partnership to boost mRNA vaccine manufacturing in Rwanda, with an aim to build a sustainable vaccine ecosystem in Africa [68]. Most recently, Gavi, the Vaccine Alliance, established the African Vaccine Manufacturing Accelerator (AVMA), which was launched in June 2024 to provide up to US$1.2 billion over the next 10 years for the development of sustainable vaccine manufacturing in Africa [69]. VacTask, an independent advisory entity, has supported South Africa as a key participant in the Africa CDC's Partnership for African Vaccine Manufacturing (PAVM), thus, uniting government ministries to align policies and regulations that support crucial aspects of vaccine-related research and development and manufacturing [70]. CEPI has also taken on an initiative to better prepare African regions for future pandemics. In West Africa, the project “Advancing Research Capacities, West Africa” (ARC-WA) has been established with the overarching aim of building clinical trials and research preparedness capacity. The IVI and the Medical Research Council Unit The Gambia at the London School of Hygiene and Tropical Medicine are joint technical coordination partners (TCPs) for the ARC-WA project. Working closely with the Africa CDC, which coordinates all pandemic preparedness activities, the TCP supports West African countries to enhance preparedness capacity for future pandemics.

Further work in administrative and regulatory aspects is needed to accelerate the development of a supportive environment. The establishment of the African Medicines Agency in 2021, the second specialized health agency of the African Union after the Africa CDC, marked an important step toward harmonization of regulatory systems for medical products across the continent [71]. Strong regulatory authorities, robust supply chains, skilled human capital, reduced trade barriers, and empowered regional coordination are needed for Africa to become more self-reliant and a global actor in the field of research and clinical development [72, 73].

## CONCLUSION

The COVID-19 pandemic has highlighted critical vulnerabilities within global health systems, particularly in Africa, as well as the continent's urgent need to invest in public health and become self-sufficient. The pandemic increased global focus on strengthening health infrastructure, investing in research and development, and fostering international and regional collaboration. Consequently, ongoing efforts are aimed at improving vaccine and pharmaceutical manufacturing capabilities in Africa. Several initiatives coordinated by the Africa CDC are underway to achieve greater African self-sufficiency in vaccine production. Continued investment, regulatory harmonization, and strengthened international and regional partnerships will be essential for Africa to develop sustainable vaccine manufacturing.

## Notes

***Author contributions.*** All authors contributed to reviewing and editing the manuscript, approved its publication, and accept responsibility for submitting for publication. Additionally, the following authors contributed to the following—conceptualization: B. T. T., F. M., J. S., T. T. T., and C. L. K.; supervision: L. M. C. E.; visualization: C. L. K. and T. T. T.; writing of the original manuscript: L. M. C. E., C. L. K., and T. T. T.

***Acknowledgments.*** The authors are grateful to Chloe Sherliker for administrative support.

***Financial support.*** This manuscript is supported by funding from The Coalition for Epidemic Preparedness Innovations (CEPI).

***Supplement sponsorship.*** This article appears as part of the supplement “COVID-19 Vaccine Research in Africa,” sponsored by the International Vaccine Institute and funded by the Coalition for Epidemic Preparedness Innovations (CEPI).
